# Opportunities, challenges and systems requirements for developing post-abortion family planning services: Perceptions of service stakeholders in China

**DOI:** 10.1371/journal.pone.0186555

**Published:** 2017-10-18

**Authors:** Hong Jiang, Jieshuang Xu, Esther Richards, Xu Qian, Weihong Zhang, Lina Hu, Shangchun Wu, Rachel Tolhurst, INPAC Consortium

**Affiliations:** 1 School of Public Health, Key Laboratory of Public Health Safety (Ministry of Education), Fudan University, Shanghai, China; 2 Shanghai Center for Women and Children’s Health, Shanghai, China; 3 Department of International Public Health, Liverpool School of Tropical Medicine, Liverpool, United Kingdom; 4 International Centre for Reproductive Health, University of Gent; School of Public Health, Université Libre de Bruxelles (ULB), Gent, Bruxelles, Belgium; 5 Huaxi Secondary Hospital, Sichuan University, Chengdu, China; 6 Clinical Research Department, National Research Institute for Family Planning, Beijing, China; National Academy of Medical Sciences, NEPAL

## Abstract

Post-abortion family planning (PAFP) has been proposed as a key strategy to decrease unintended pregnancy and repeat induced abortions. However, the accessibility and quality of PAFP services remain a challenge in many countries including China where more than 10 million unintended pregnancies occur each year. Most of these unwanted pregnancies end in repeated induced abortions. This paper aims to explore service providers’ perceptions of the current situation regarding family planning and abortion service needs, provision, utilization, and the feasibility and acceptability of high quality PAFP in the future. Qualitative methods, including in-depth interviews and focus group discussions, were used with family planning policy makers, health managers, and service providers. Three provinces—Zhejiang, Hubei and Yunnan—were purposively selected, representing high, medium and relatively undeveloped areas of China. A total of fifty-three in-depth interviews and ten focus-group discussions were conducted and analysed thematically. Increased numbers of abortions among young, unmarried women were perceived as a major reason for high numbers of abortions. Participants attributed this to increasing socio-cultural acceptability of premarital sex, and simultaneously, lack of understanding or awareness of contraception among young people. The majority of service stakeholders acknowledged that free family planning services were neither targeted at, nor accessible to unmarried people. The extent of PAFP provision is variable and limited. However, service providers expressed willingness and enthusiasm towards providing PAFP services in the future. Three main considerations were expressed regarding the feasibility of developing and implementing PAFP services: policy support, human resources, and financial resources. The study indicated that key service stakeholders show demand for and perceive considerable opportunities to develop PAFP in China. However, changes are needed to enable the systematic development of high quality PAFP, including actively targeting young and unmarried people in service provision, obtaining policy support and increasing the investment of human and financial resources.

## Introduction

Although there is limited evidence on the health risks of repeat abortions, there is some evidence of increased risk of reduced fertility, complications and poor outcomes in future pregnancies, in addition to potential psychological impacts [1–4]. It was estimated that 43.8 million abortions occurred worldwide in 2008, among which 41% were unintended [5,6]. In China, 13 million induced abortions were conducted in 2008, according to the latest available figures [7]. A cross-sectional survey of hospital data from 30 provinces in 2013 found that of 79,174 women seeking abortions within 12 weeks of pregnancy, 37% were undergoing a second abortion, and 29% a third or subsequent abortion [8].^.^ This suggests that whilst access to safe abortion is a critical reproductive right, preventing unintended pregnancy where possible through access to high quality contraceptive services is also important to promote women’s health, particularly in future pregnancies. As one type of family planning service, post-abortion family planning (PAFP) services aim to “assist women to identify the family planning problems leading to the unintended pregnancy, and help them to develop strategies to prevent their re-occurrence” [9,10]. PAFP has been proposed as a key strategy to decrease unintended pregnancy and repeated induced abortions [9, 10]. There is evidence that post abortion contraception acceptance rates increase from under 10% to 50–80% within one to two years if PAFP is offered immediately after the abortion and before a woman leaves the facility [11, 12]. However, the accessibility and the quality of PAFP services remains a challenge in many low and middle income countries including China.

Induced abortion was legalized in China as early as 1953 and abortion services became widely available following the launch of the Family Planning Policy in 1973 [13]. The induced abortion rate reached its highest level in the early 1980s (56.07%) and its lowest level in the 2000s (18.04%). However, abortion incidence appears to have risen in China since 2003 [5]. According to available evidence, unintended pregnancies account for the majority of abortions [14, 15]. Around half of unintended pregnancies are attributed to contraceptive failure (40–56%) and the other half to non-use of contraceptives (44–50%) [7, 16–20].

Since China’s economic and social reform in the 1980s, people’s perspectives and attitudes towards sex and abortion have changed significantly, with increasing acceptance of premarital sex and extramarital sex [21, 22]. However this has not been accompanied by a corresponding improvement in contraceptive knowledge and utilization, especially among young people [23]. The official age of marriage in China is 20 for women and 22 for men [24]. Traditional social-cultural norms and taboos regarding premarital sex and pregnancy limit the friendly provision of family planning services to unmarried young people and prevent from seeking help regarding contraceptives [25, 26]. The profile of women utilizing abortion has shifted toward younger women, at a rate beyond the change in composition of the population as a whole. Cross-sectional facility data from 30 provinces in 2013 found that 31% of abortion service users were unmarried [4]. Some studies have shown very low knowledge of and utilization of contraception amongst teenage women seeking abortion [27].

Furthermore, with rapid urbanization, young rural-to-urban migrant women are also a social group increasingly represented amongst women seeking abortions. A survey among over 18,000 women undergoing induced abortion due to unintended pregnancy in Beijing in 2010–11 found that migrants accounted for the majority of the abortion service users [28]. Similarly, facility data from 30 provinces in 2013 found that 61% did not have local residency status [4]. Overall, this group tends to be relatively uneducated, in low-income and insecure jobs, lacking health insurance coverage and excluded from municipal welfare structures [28]. Contraceptive use rate is high among married women but the need is increasing amongst unmarried women [29] High risk and vulnerable groups among the unmarried, such as young women and rural-to-urban migrant women often face unmet family planning needs.

In China, the vast majority of induced abortions are performed in public hospitals, although family planning clinics are also able to provide abortion services [30]. Since 2010, 486 hospitals in China have been included in a project to pilot provision of PAFP services, generally known as ‘the PAC project’ [31]; however this is a very small proportion of a total of more than 50,000 hospitals [individual communication] who provide abortion services nationally and has not been rigorously evaluated. PAFP service provision has not yet been integrated into the Chinese health system. The majority of women seeking abortion therefore have no access to PAFP. Although a number of studies have called for the development of PAFP services [1, 16, 17], few of them have assessed the opportunities and explored the barriers and system requirements for integrating the provision of a high quality service into general health facilities. This is of particular importance when considering the sexual and reproductive health needs of vulnerable and marginalized groups such as young people, migrants and those of ethnic minorities, both in China and in LMICs [26, 32].

The “Integrating Post-Abortion Family Planning Services into existing abortion services in hospital settings in China” [INPAC] project [33], aimed to evaluate the effect of integrating PAFP services into existing hospital based abortion services in China, by developing, implementing and evaluating a uniform intervention, which was separate from the PAC pilot study. This paper presents data collected as part of the INPAC baseline, which aimed to contribute towards the development of the intervention by exploring the perceptions of the key service stakeholders [policy makers, managers, service providers] on the current situation regarding family planning and abortion service needs, provision, utilization, and the feasibility and acceptability of PAFP in the future. Perceptions and experiences of service users were also sought and are reported elsewhere [34].

## Methods

### Setting

Three provinces–Zhejiang (ZJ), Hubei (HB) and Yunnan (YN)—were purposively selected, representing high, medium, and relatively undeveloped areas of China [35]. Within each province, one city and one rural county were selected, with representation across the sample of: relatively rich and poor cities/counties; a high population proportion of ethnic minorities (one county); and high levels of rural-to-urban migration (one city). In each city, two facilities were selected: one secondary-level hospital (medium sized hospital) and two tertiary-level hospitals [one general or comprehensive hospital at city level and one maternal and child health hospital at city level]. In each rural county, two facilities were selected: one primary-level hospital (township hospital), and one secondary-level hospital (county level). In Zhejiang province, in the rural setting, one maternal and child health institute and one secondary-level hospital were selected as these two institutions provide the majority of abortion services in the area. Service stakeholders eligible for participation in the study were defined as 1) health service managers and providers directly involved in providing or managing abortion or post-abortion care in the selected health hospital/health institutes; 2) Policy makers at provincial, prefecture and county levels, including officials responsible for abortion services at the Public Health Bureau and at the Population and Family Planning Commission.

### Study design

Qualitative methods including semi-structured interviews and focus group discussions were used to explore the views of key service stakeholders in relation to their specific context. Participants were purposively sampled to include informants with first-hand experience of making provincial or county policy, or managing and/or providing abortion services. Semi-structured interviews were used to explore individual experiences and perspectives from different types of participant, whilst focus group discussions aimed to enable providers to discuss commonalities and areas of disagreement as well as identify public narratives about abortion and family planning [36].

### Ethical considerations

Written informed consent was obtained from each participant before the interview/discussion was held. The research was approved by the Liverpool School of Tropical Medicine Research Ethics Committee, UK and institutional ethics boards in China, including: the Institutional Review Board of the School of Public Health, Fudan University, Shanghai, and the Chongqing Medicine University Ethics Committee.

### Data collection

Before the interviews, the topic guides for different stakeholders were developed and piloted. Potential respondents were contacted by the local health staff via telephone in advance, to verify their eligibility and arrange for an interview time and the place. The interviews were carried out face to face in a private room after obtaining informed consent. All interviews were conducted in Mandarin Chinese. In each province, two trained researchers conducted the interviews, one as the facilitator and another as the recorder and note taker. All qualitative interviews lasted between 30 to 90 minutes and were digitally recorded. Interviews ceased when saturation was reached.

### Data analysis

All the interviews were transcribed ‘verbatim’ into Mandarin Chinese and kept as Microsoft Word documents. Data were analysed using a framework approach and were initially analysed based on province unit using a common framework. In each province, two researchers carefully read and coded all transcripts separately. Then they compared and discussed differences in coding until agreement was reached. A node tree was devised and used to classify and organize data according to the framework using Nvivo 7.0 computer software. The three teams who collected data in each province discussed the coded data, identified themes through rounds of discussion and translated themes and relevant raw data into English (by HJ & JXU, who are native Mandarin Chinese speakers with fluency in English and experience in translation). Finally, all data were brought together and discussed in a workshop to enable comparison and contrasting of data and joint analysis [37]. Table 1 presents the final categories, sub-themes and themes emerging from this process.

**Table 1:** Analytical framework development.

Main theme 1: Perceptions of the need for PAFP		
Sub-themes:		
High number of abortions among young, unmarried women	Gaps in contraceptive information and provision [for marginalised groups]	
Codes: **changing sexual behaviour; information sources on FP; provision of FP services; limitations of FP services; accessibility of FP services; utilisation of FP services; need for improvements to FP services; abortion acceptability; reasons for unintended pregnancy; reasons for abortion; consequences of abortion;**		
Main theme 2: Perceptions of current PAFP provision and potential for future implementation		
Sub-themes		
Extent of PAFP provision is varied and limited	Positive perceptions of PAFP in principle	Providing PAFP is challenging
Codes: **perceptions of PAFP; perceptions of extent of PAFP provision; potential for developing PAFP; opportunities for developing PAFP**		
Main theme 3: Health systems considerations for implementing PAFP		
Sub-themes:		
The need for a policy support	Financial and human resource considerations	
Codes: **Constraints to developing PAFP; potential impact of developing PAFP; resources needed to develop PAFP; who should provide PAFP; potential organisation of PAFP**		

## Results

From March to July 2013, a total of fifty-three in-depth interviews (IDIs) and ten focus-group discussions (FGDs) were carried out in three provinces. Twenty- seven interviews were carried out with health policy makers at provincial, urban district level and rural county level. Ten FGDs were carried out with health managers, and abortion service providers at the selected hospitals. Fifteen interviews were also held with hospital and family planning managers at each hospital and eleven interviews were conducted with service providers to triangulate data produced in the FGDs (Table 2).

**Table 2:** Data collected across all three provinces.

Method and participant type	Province (study site level)	Total number
Key informant interviews with policy makers (27)	Hubei	9
	Provincial	3
	Urban (prefecture)	3
	Rural (county)	3
	Yunnan	10
	Provincial	4
	Urban (prefecture)	3
	Rural (county)	3
	Zhejiang	8
	Provincial	3
	Urban (prefecture)	2
	Rural (county)	3
In-depth interviews with abortion service managers (15)	Hubei	4
	Urban	2
	Rural	2
	Yunnan	4
	Urban	2
	Rural	2
	Zhejiang	7
	Urban	3
	Rural	4
In-depth interviews with service providers (11)	Hubei	4
	Urban	2
	Rural	2
	Yunnan	4
	Urban	2
	Rural	2
	Zhejiang	3
	Urban	3
	Rural	0
Total interviews		53
FGDs with abortion service providers	Hubei	3
	Urban	2
	Rural	1
	Yunnan	3
	Urban	2
	Rural	1
	Zhejiang	4
	Urban	2
	Rural	2
Total FGDs		10

### Perceptions of the need for PAFP

#### Abortion seems popular among young, unmarried women

Abortion numbers were perceived as high by all participants, who thought this was linked to changes in socio-cultural norms. In particular, an increase in abortions among young, unmarried women was seen as a major reason for high numbers of abortions. This was attributed by the majority of participants across all three provinces, in rural and urban areas, to the increasing acceptability of premarital sex as part of changing social attitudes:

ZJ People are very casual towards premarital sex. It is very common today and does not attract people’s attention. In particular, parents today are not as strict as our parents in old days. Premarital sex is more acceptable today than before. This is certainly a sign of social development.

(IDI with Policy maker, HB province)

Participants, particularly in YN and ZJ province, saw pre-marital sex as leading to abortion due to young, unmarried people being less likely to use contraception. Lack of understanding or awareness of contraception was one of the main perceived reasons for this:

The younger the age is, the less likely they will protect themselves, especially for students or those under 20—situations get a little better for those above 25.

(IDI with manager, YN province)

Most couples use emergency contraceptives because they fear being pregnant… Most of them [young unmarried women seeking induced-abortion] did not use any contraception method. They always think they would not get pregnant.

(FGD with FP service providers, ZJ province)

Some participants also perceived young people to have ‘casual’ attitudes towards abortion, asserting that young people use abortion as a contraceptive method. Some health managers in HB and YN felt that the rise in abortion rates was not acceptable and a number of service providers from all provinces perceived an increase in ‘misleading propaganda’, for example in relation to advertisements for abortion under anaesthetization:

Many private hospitals, TV programs and newspapers over-advertise ‘painless abortion’ which misleads many young people. Young people think that it only takes 3 minutes and it’s simple without any pain. They regard it as a “kind” of contraception.

(IDI with health manager, HB province)

So easy! All that you need to do is a short sleep!

(IDI with service provider, YN Province)

Furthermore, participants across all provinces perceived that abortions were common among rural-to-urban migrants, the majority of whom are relatively young, and unmarried. Low educational level was also an important perceived reason for lack of knowledge on contraception.

Unmarried people, especially the young people, are the main abortion users. Among those unmarried people there are a large number of internal migrants.

(FGD with health service providers, ZJ Province)

#### Gaps in contraceptive information and provision [for marginalised groups]

The majority of participants across all three provinces perceived family planning services as widely accessible. Services described included the availability of a range of contraceptive methods at ‘family planning stations’ and hospitals, including IUD insertion and removal, condoms, contraceptive pills, and male and female sterilization. Other types of contraception were sometimes reported as being available at family planning stations (such as Norplant) or at tertiary hospitals (such as contraceptive foam). The majority of service stakeholders across all three provinces reported that these services were available free of cost or reimbursable through health insurance to married couples, but that services were limited for migrants, young, unmarried people and ethnic minorities.

Most participants in all provinces acknowledged that the majority of family planning services are neither targeted at, nor easily accessible to unmarried people. Oral contraceptives and/or condoms were said by some to be available without charge for unmarried people at health facilities, and condoms were reported to be distributed for free to migrant workers by some participants (e.g. in HB), but the availability varied within and between provinces. Furthermore, payment is required for all other services.

For married people, they mainly access contraception through hospital and pharmacy. I’m not quite familiar with unmarried people. I think it will be more difficult considering the tradition and psychological issues……For the migrant population, it will be more difficult compared with local residents. I think they don’t know where to get contraception.

(IDI with health manager, HB Province)

Some service stakeholders, across all three provinces, emphasized the lack of effective contraceptive education provided to young people either from their families or through their schools. Participants perceived that family planning education in middle schools was discouraged by the authorities:

When we communicate with the young junior middle school students, we find that they are completely confused about knowledge of contraception and their parents can’t find an appropriate way to communicate with them. … No media introduces correct methods of contraception to the public. Free contraceptive pills or condoms are mostly provided for married people by Family Planning institutes. In fact, it is very important to provide Family Planning services for the unmarried people, because there are a lot of complications of abortion, which are likely to affect their fertility in their future life.

(FGD with health service providers, ZJ Province)

In HB and YN province some service stakeholders mentioned that married women are usually targeted for services, and some identified that this leads to a problematic lack of focus on men’s responsibilities:

Most male partners think contraception is women’s responsibility. It’s not their problem.

(FGD with health service providers, HB Province)

Actually it is more important to carry out education for men, since in many cases, [unintended pregnancy] results from men’s unwillingness to use condoms.

(IDI with health manager, YN Province)

In YN Province, some participants perceived difficulties experienced by ethnic minorities in accessing services related to communication with providers, especially for older people who are less likely to speak standard Mandarin.

It's difficult to communicate with ethnic minorities. Young people are OK—they have basically completed secondary education—yet those above 40 are less likely to speak mandarin. There are 13 ethnic minorities YNhere, and they speak different dialects.

(IDI with manager, YN province)

Distance to family planning stations for remote rural populations was also seen as a physical barrier to accessing services. A minority of policy makers also mentioned a lack of FP services for HIV positive women and lack of choice of contraceptive methods for service users.

### Perceptions of current PAFP provision and potential for future implementation

#### Extent of PAFP provision is varied and limited

Participant views on the current extent of PAFP provision varied between provinces. For example service stakeholders from HB perceived that PAFP services exist fairly widely with most facilities providing some form of family planning information as part of abortion services. In ZJ, FP service providers from an urban tertiary hospital reported they have been trained for PAFP and perceived that they are providing the service well. However, participants reported that PAFP is not available in urban secondary hospitals. FP service providers in rural ZJ regarded themselves as providing PAFP services; however, they were not able to describe a standardized procedure for PAFP service provision. No participant from YN reported PAFP provision in rural areas.

Our hospitals mainly focus on medical services. Doctors do not provide much health education and consulting in the hospitals. After surgery, we mainly inform patient about the medical issues, [and say] little about contraception.

(IDI with manager, ZJ Province)

The majority of participants in YN and ZJ and a minority in HB perceived current PAFP services provided as limited. These perceived limitations were due to lack of detailed national guidelines for PAFP, lack of motivation among FP service providers, lack of financial and human resources, and limited time commitment for following up of service users. These issues are described more detail below.

#### Positive perceptions of PAFP in principle

In HB and tertiary hospitals of urban ZJ, almost all participants among policy makers, managers and service providers mentioned that there are currently positive attitudes among doctors towards PAFP. These were framed in terms of both willingness and a sense of duty. Support was also perceived from hospitals, some of which are already engaged in providing some of form of post abortion care [PAC].

A number of these service stakeholders also perceived that the potential positive impacts would include social benefits, such as a reduction in numbers of abortions and an increase in women’s awareness of how to protect themselves. In HB province, a range of potential institutional benefits were perceived by policy makers, managers and service providers including improvement of technical skills, improvement of services, and enhanced safety of services through standardization of PAFP. Some HB facility managers mentioned that these benefits could potentially enhance hospitals’ reputations, which could in turn attract greater number of patients and therefore lead to increased income at those hospitals.

In 2011, we started systematic and standard PAFP. We allocated a special consultation room for PAFP. Before we also provided a similar service but at that time there was no specific term like ‘PAFP’… The core idea of PAFP is care after abortion that does not simply focus on abortion, but emphasizes contraceptive use after the abortion to prevent repeated induced abortion.

(FGD with service providers, HB Province)

#### Providing PAFP still faces challenging

However, some service providers in HB and ZJ Provinces discussed their experiences of the challenges of providing PAFP, reporting that some clients did not ‘comply’ with advice given; clients were characterized as lacking in knowledge and understanding. Unmarried women were seen as particularly difficult to contact for follow-up counseling:

We feel it is hard to reach the target of [successfully following up] 80% [of women after abortion]. Lots of women give a false name and a false telephone number to us. They do not want their boyfriend to know [about the abortion] or it was due to an extra-marital affair… however, we are still carrying out follow-up, although we are sometimes blamed by the clients.

(IDI with service providers, ZJ Province)

In addition, the institutional and cultural confusion about how to ‘manage’ sexually active young people was expressed by most participants of three provinces in discussing the challenges of carrying out PAFP. Participants expressed unease about discussing contraceptives with young people and many did not view this as primarily the responsibility of abortion-service providers, emphasizing that more contraceptive education was required at schools.

If it is a married woman of childbearing age, which means she has a family and insurance, then you can implement PAFP according to the requirements. She should be provided with instruction, I agree, and the effect [of information on conceptive use] will be better. But if it is a student standing before you, how you are supposed to provide instruction? Are you going to give her a scolding or look down upon her? What kind of method is good for her? How to change her behavior and improve her health? We have no idea at present!

(IDI with policy maker, YN province)

It is not only our responsibility [to provide reproductive health education to young people]… Education should be provided before sexual behavior happens… School should give reproductive health education to students in junior middle school or even earlier.

(FGD with service providers, ZJ Province)

### HBHealth systems considerations for implementing PAFP

Service stakeholders across all provinces described three main considerations regarding the feasibility of developing and implementing PAFP services: policy support, human resources, and financial resources.

#### The need for a policy support

Many policy makers and service providers in all three provinces emphasized the need for policy support. A common perception amongst service stakeholders was that policy support for PAFP services would be needed in the form of regulation and guidelines mandating their provision. Both service providers and policy makers expressed the need to include PAFP service delivery in the indices used by the Health Bureau to assess facility performance.

We will assess the work plan and the feasibility for relevant hospitals. I think if possible we can add content of PAFP services to their work plans, and evaluate at the end of every year.

(IDI with policy maker, HB province)

There must be a policy to include PAFP services as part of assessment content. Only doing this can PAFP services become routine work.

(FGD with service providers, ZJ Province)

[If there is] no policy, [then there are] no guidelines and no assessment. The hospitals don’t have motivation as well as pressure to provide PAFP.

(IDI with policy maker, HB province)

A number of service stakeholders also perceived the need for more detailed clinical guidelines and standards and a clear regulation structure to accompany policy, to address concerns about assuring the quality of services.

Policy makers and service providers from all three provinces referred to the merging of the Ministry of Health and the Family Planning Commission (MHFPC) as an important opportunity for developing PAFP services. It was suggested that these previously separate institutions could now work together more effectively in this area by playing complementary roles; for example FP institutions could work on organization, awareness raising and follow-up, whilst health institutions could provide technical expertise. Some participants linked the opportunity to the possibility of sharing resources and the fact that the FP sector was fully funded by the government in contrast to health facilities, which receive only limited government funding and are therefore required to generate income through service provision.

The state population and family planning committee has been merged with the ministry of health. This will provide us some opportunities. At least, this is beneficial for the integration of family planning service provision. In the near future, township and village family planning stations or family planning offices can provide free contraceptives, and our health facilities can integrate this into our services and enhance the content of our services. I do think it’s feasible.

(IDI with policy maker, ZJ Province)

#### Financial and human resource considerations

In addition to the importance of a policy mandate the majority of service stakeholders stressed that the financial implications of developing PAFP services need consideration. Although one manager anticipated that improvements in service quality through extending PAFP would actually increase institutional earnings due to attracting more patients, some service providers and managers feared that implementing PAFP would ultimately lead to a reduction in abortion service volume, which would reduce their own earnings, since these are linked to hospital income generation.

If the quality of service is improved, [the hospital] will attract more patients. When the service quality becomes better, institutional earnings will be naturally increased.

(IDI with hospital manager, HB Province)

Our service providers think PAFP is a burden. First it will increase workload, and this kind of workload is not related to economic benefit. Second, if PAFP is effective, the amount of abortions will decrease and our salary will decrease because the health system [here] is mainly a market economy. That’s why we don’t really care about this PAFP……There isn’t any support in terms of policy, technology, and finance. China medical association came to develop the guideline, but they didn’t provide financial support.

(IDI with hospital manager, HB province)

The majority of service providers in all three provinces perceived that expansion of PAFP services would substantially increase their workloads and saw this as a likely obstacle to the feasibility of implementation. Those already providing PAFP reported that it had led to increased workloads:

Participant a: [Since the provision of PAC,] workload is larger.

Participant b: Our work is more intensive than before. It makes me busy. We have to tell them [about conceptive use] before the abortion and also after the abortion, plus follow-up.

(FGD with service providers, ZJ Province)

Similarly the majority of hospital and departmental managers and policy makers perceived the need for increased human resources to provide PAFP services.

There must be human resource quotas for PAFP services. The hospital should give our department as least 2 staff members that focus on provision of PAFP services. If we have these 2 people, the hospital will give us financial support for their salary. Free PAFP services will certainly increase our burden. This thing is not difficult to implement with human resources and funds.

(IDI with service manager, YN province)

Another issue mentioned by a number of service stakeholders in all three provinces was the importance of motivating providers through an incentive structure. This was expressed in slightly different ways across different service stakeholder groups: for example, some stated the need for PAFP to be included in the set of performance indicators for individual providers within facilities and thus linked to income. Others perceived the need for improved pay.

Many stakeholders, particularly policy makers and managers, were also concerned about skills and training for abortion service providers, both in technical knowledge on FP and communication and counselling skills, and the availability of appropriate educational materials to give to clients. However, some health managers in HB referred to PAFP as “not technically difficult”.

Hospital service providers focus on clinical services. Now they have to provide individualized counselling, their interpersonal communication skill should be improved. They need training.

(IDI with policy maker, ZJ Province)

Finally, policy makers and managers in HB and YN provinces also mentioned the importance of physical spaces dedicated to the provision of PAFP services, though some saw these as not essential:

It will be best if special IEC is done in a dedicated venue, but it requires provision of both human resource and funding. However, when such conditions are not matched, a conscientious doctor who is trained sufficiently in this aspect can do it well too, even if there is no dedicated venue.

(IDI with service manager, YN province)

## Discussion

The perceptions of service stakeholders including health policy makers, managers and service providers, suggest a number of important areas for attention in the development of equitable, sustainable and effective post-abortion family planning services.

Service stakeholder perceptions that abortion rates are relatively high, particularly amongst young unmarried women and rural-to-urban migrants are consistent with the description of the current abortion rates [1, 38, 39] and the [over-] representation of these groups amongst abortion service users [28, 40, 41]. The view that rapid social change underlies this, including change in sexual norms and behaviour, also concurs with explanations offered for China’s increasing abortion rates in the international literature [1, 14]. Some stakeholders revealed ambivalent attitudes towards the sexuality of young, unmarried people, who were simultaneously characterised as ‘careless’ and also as victims [for example of misleading advertising of abortions]. Such ambivalent attitudes towards adolescent sexuality and abortion use have been found amongst service providers in China and internationally [42, 43]. Globally, young people are often implicitly or explicitly blamed for their use of abortion, despite their poor access to contraceptive information and services [44]. However, service providers have also simultaneously demonstrated empathetic and pragmatic attitudes towards young people seeking abortion [44].

In general stakeholders perceived FP services as highly accessible, cohering with high reported rates amongst married couples [45]. Many were clearly cognisant of the limited access to information and services for young unmarried people, and rural-to-urban migrants; a number of participants stressed the lack of school-based contraceptive education as a key issue. However, few participants discussed the need for youth-friendliness in PAFP services. An exception was a participant who stressed the lack of guidance and skills to support providers to give effective PAFP counselling to young, unmarried people in the context of ambivalence towards their sexual activity. ‘Youth-friendly’ family planning requires specific attitudes and skills, which are not explicitly fostered in the current context or included in standard counselling approaches in China. The findings indicated the current sexual and reproductive service provision in China does not address young people’s concerns and needs, and on the contrary, young people were implicitly blamed for lacking knowledge and awareness of protecting themselves. This was consistent with other international studies that also reported unfriendly or judgemental attitudes from health staff [46, 47]. Youth-friendliness of reproductive health services has remained poor in many settings despite two decades of global motivation on removing barriers for adolescents’ access to services [48, 49]. Both the World Health Organization (WHO) and PAC consortium identify differences in the needs of young people (from 15 to 24 years old) as compared to older women as PAC service clients [42, 50]. WHO PAFP guidelines include consideration of the specific needs for adolescents [9], and emphasize the importance of providing them with access to counselling and contraceptive options that maximize protection against unintended pregnancy [9]. PAFP has also been identified as a cost-effective way to reach young people who have unmet needs for contraceptive information and services [10, 50]. Specific acknowledgment of the needs of young, unmarried people will be needed to ensure that PAFP services meet their needs, including explicit attention to discussing ambivalent attitudes towards their sexuality and developing a non-judgemental stance in counselling. Schools’ roles in reproductive health education should also be strengthened. Training to develop critical thinking on the cultural and ethical dimensions attached to adolescent sexuality may be effective here [44, 47].

Some service stakeholders perceived difficulties for migrants to access Family Planning services, due to non-transferrable health insurance and different welfare policies between provinces. Efforts from national and provincial levels are needed to address the barriers to FP services for rural-to-urban migrants, especially with policy makers. One participant’s description of young, unmarried migrants as ‘unrestrained’ in comparison with ‘our local’ young people, also hints at the marginal and ‘outsider’ status of this group, and suggests the need to develop neutral and non-judgemental attitudes amongst service providers. In YN province some providers discussed the problems in communication with older clients from ethnic minorities due to language barriers; efforts to overcome such barriers need to be made in areas with large ethnic minority populations. A minority of participants raised the need to further include and target male partners in the provision of both Family Planning and PAFP services. Internationally, there is a general consensus on the importance of male involvement in reproductive health, including family planning programmes. A number of studies internationally suggest the influence of male participation on women’s use of contraceptives [51, 52].

The importance of a policy regulation, as emphasised by the service stakeholders, supports international evidence for the importance of ensuring a supportive policy environment through either developing PAC/PAFP service guidelines and or new national policies incorporating PAC/PAFP into reproductive health services to the scale-up of PAC/PAFP services [53]. Policy support and financial resources are the two necessary components in WHO's health system strengthening blocks [54]. Furthermore, WHO published detailed guidelines for PAFP in 1997 [9], but these were not immediately applied in China. Until 2011, national PAFP guidelines [55] were issued by the Family Planning Association, which is a branch of the China Medical Association. However, these provided no detailed steps for PAFP service provision. Within the highly hierarchical Chinese health system, detailed policy provisions are needed to ensure the systematic implementation of PAFP.

Service stakeholders further stressed the importance of sufficient resource allocation at both institutional and individual levels. The inclusion of a specified service process aiming at different social groups and expected service components to be provided by different health actors is required to enable and guide resource allocation to the corresponding sectors. Within a highly commodified health system that motivates providers through financial incentives linked to institutional and individual assessment criteria, the integration of PAFP services into performance indicators is critical.

Participants generally expressed a certain level of intrinsic motivation to provide PAFP services, related to their perception of the possible benefits of service provision. However, many recognised the need for further training in both technical and communication skills to enable the provision of high quality PAFP services. This supports another recent study that found demand from service providers for training on PAFP counselling and contraceptive methods in Shanghai [56]. A review of 28 studies of PAC scale-up in 20 different countries found that training of a range of service providers was a critical common element [57]; a focus on both in-service and refresher training is important to ensure sustained high-quality service provision [10].

This is one of the very few studies to explore service stakeholder perceptions of post-abortion family planning in China; none have been published in English language journals. Its strengths include our in-depth qualitative approach, which allowed concerns of the relevant stakeholders to emerge, and the maximum variation sampling approach which enabled symbolic representation of key diversities in the Chinese context, including regional income levels, ethnic diversity, rural; and urban, migrant and stable populations, as well as different levels and types of facilities: Although thematic saturation was reached, this relatively small sample cannot be considered representative of all contexts within China. Since researchers were associated with the wider INPAC project, this may have influenced service stakeholders’ expressions of support for PAFP, although their candour on the potential challenges is reassuring. Another potential limitation is loss of meaning in the translation process; this risk has been mitigated to some extent by analysing all data in Chinese before translating identified themes into English for discussion. Further research is needed to explore service stakeholders’ perceptions and experiences of sustained, systematic implementation of PAFP services.

## Conclusion

Service stakeholders in the three diverse provinces of China generally agreed on the need for post-abortion family planning services and identified key population groups at particular risk of unintended pregnancy. In common with other contexts, including low and middle income settings, the sexual and reproductive health needs of vulnerable and marginalized populations require particular attention. Fostering non-judgemental and sensitive attitudes towards adolescent sexuality and rural-to-urban migrants and developing appropriate health promotion approaches for these groups should be considered in provider training. Based on current health system functioning, systematic changes are needed to develop high-quality PAFP services, including detailed PAFP service guidelines and standards, financial and human resources investment, performance assessment mechanisms and training for providers.

## Abbreviations

FDG: Focus-group discussion
IDI: In-depth interview
INPAC: Integrating Post-Abortion Family Planning Services into existing abortion services in hospital settings in China
MHFPC: Family Planning Commission
PAC: Post-abortion care
PAFP: Post-abortion family planning
WHO: World Health Organization
